# Primer on adult patient satisfaction in perioperative settings

**DOI:** 10.1186/s13741-019-0122-2

**Published:** 2019-09-19

**Authors:** Lily N. Trinh, Michelle A. Fortier, Zeev N. Kain

**Affiliations:** 1 Center on Stress & Health, University of California School of Medicine, Irvine, USA; 2 Sue & Bill Gross School of Nursing, University of California, Irvine, USA; 3 Department of Anesthesiology and Perioperative Care, University of California, Irvine, USA; 4 Yale Child Study Center, Yale University, New Haven, CT USA; 5 Health Policy Research Institution (HPRI), University of California, Irvine, USA

**Keywords:** Adult patient satisfaction, Perioperative, Review, Clinical communication, Information provision

## Abstract

The topic of patient satisfaction has gained increasing importance over the past decade. Due to the impact of patient satisfaction on health care quality, understanding factors that predict satisfaction is vital. The purpose of this review is to examine the literature and identify factors related to patient perioperative satisfaction as well as predictive variables that, if modified, can enhance satisfaction scores of patients undergoing surgery. Our review reports that patient satisfaction scores are affected by modifiable factors such as clinician-patient communication, information provision to patients, and operational function of a hospital. Non-modifiable factors affecting patient satisfaction scores include patient demographics such as gender, age, and education. In order to enhance patient perioperative satisfaction, we suggest that anesthesiologists and surgeons focus their efforts on enhancing their communication skills and providing information that is appropriately tailored to the understanding of their patients.

## Background

In 2016, the United States (U.S.) spent nearly 18% of its gross domestic product (GDP) on healthcare whereas the next highest comparable country (Switzerland) devoted less than 13% to this category (OCED, 2018). Within that context, the U.S. also ranks as one of the worst in health care parameters such as infant mortality and prevalence of chronic diseases (Squires & Anderson, 2015). The high U.S. health care system GDP, which has been fueled for decades by increased operational costs, is mostly without corresponding improvement in clinical outcomes (Obama, 2016). In an effort to improve health care outcomes and decrease cost, the U.S. government has adopted a series of measures that are based partially on the Triple Aim proposed by Don Berwick: (1) improve patient experience, (2) improve population health, and (3) reduce per capita healthcare costs (Berwick et al., 2008).

Based on the work of Berwick and others, in 2013, the Centers for Medicare and Medicaid Services (CMS) initiated the hospital Value-Based Purchasing (VBP) program that altered hospital reimbursement from procedure-based to hospital performance data. This novel approach has challenged medical institutions to deliver high-quality care and reduce costs (Haley et al., 2016). As of 2018, there are four equally scored domains of a hospital’s VBP score: safety, clinical care, cost reduction, and patient experience. Because 25% of a hospital’s VBP score for 2018 is measured by patient experience, hospitals have been increasingly investing in strategies to improve patient-related experience and satisfaction (Kain et al., 2014; Dalal et al., 2016). Accordingly, this growing focus towards evaluating patient experience outcomes should prompt health care institutions to explore critical factors that may advance overall health outcomes and care.

**Fig 1:**
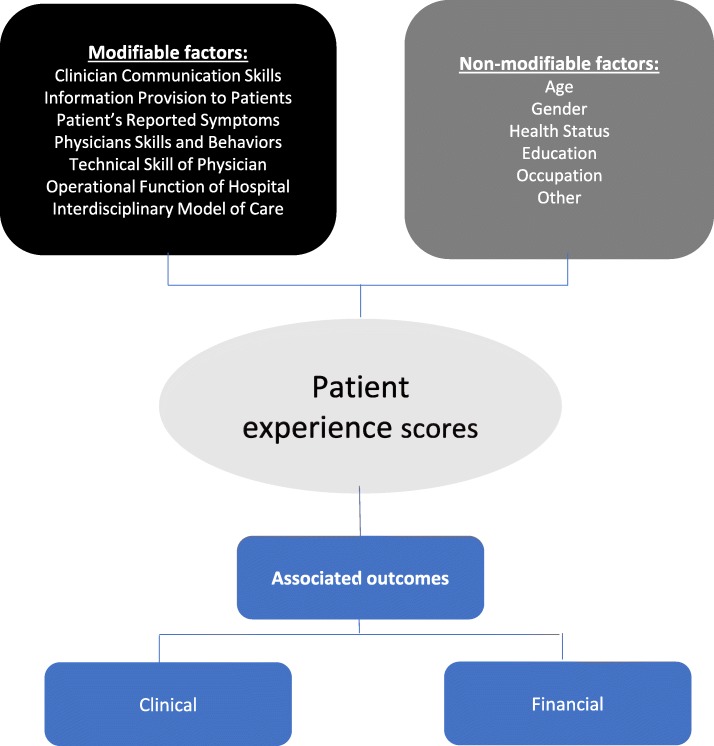
Flowchart of factors influencing patient experience scores and its associated outcomes. Predictors of patient satisfaction scores and its associated outcomes

We submit that because healthcare organizations are increasingly emphasizing patient satisfaction, it is imperative that anesthesiologists and surgeons be educated on the concept of patient experience within the context of the perioperative environment. The purpose of this report, therefore, is not to merely review the topic of perioperative patient satisfaction, but rather focus on the identification of several variables that have been identified in the literature as predictors of satisfaction scores (Fig. 1). Indeed, while many anesthesiologists and surgeons believe that patient satisfaction with their perioperative experience is a function of technical variables such as surgical and anesthetic techniques, in reality, patients do not have access to such information and as such rely solely on surrogate variables such as communication skills and empathy of perioperative clinicians.

### Hospital patient satisfaction measurement: a brief history

In 1995, the Center for Medicare and Medicaid Services (CMS) partnered with the Agency for Healthcare Research and Quality (AHRQ) to develop the Consumer Assessment of Health Providers and Systems (CAHPS) surveys in response to the lack of available information regarding consumers’ experiences of their health care and services (CMS. Center of Medicare and Medicaid Services, 2018; Goldstein et al., 2005). The goals of the CAHPS surveys are to (1) develop standardized surveys that organizations can use to collect comparable information on patients’ experience of care and (2) generate tools and resources to support the dissemination and use of survey results to inform the public and improve health care quality (CMS. Center of Medicare and Medicaid Services, 2018; Agency for Healthcare Research & Quality, 2018). The hospital-specific survey, Hospital Consumer Assessment of Healthcare Providers and Systems (HCAHPS), was implemented nationally in 2006 and includes questions on communication with nurses and doctors, the response of hospital staff, hospital environment, communication about medicines, discharge information, overall hospital rating, and patient demographics.

The first public reporting of HCAHPS data was available in 2008 and included 4032 hospitals. A *New England Journal of Medicine* study using these data showed that on average, 63% of patients rated their care greater than 9 out of 10, 26% gave a rating between 7–8, and only 11% gave a rating less than 6 (Jha et al., 2008). Since the first public reporting, patient satisfaction scores have increased in all categories, although it is unclear if patients are more satisfied with their care or hospitals are better at managing the episode of care based on the HCAHPS questions (Mann et al., 2016).

### Understanding patient satisfaction

Patient satisfaction is a subjective, complex, and multi-dimensional measure. It is defined as a health care recipient’s evaluation of the care they received and is affected by the recipient’s expectations and outcomes (Pascoe, 1983). Dissatisfaction occurs when discrepancies exist between a patient’s experience and their expectations. Given the multi-faceted nature of satisfaction including physical, emotional, social, and cultural components, it is no surprise that patient satisfaction is difficult to measure. While many validated patient satisfaction questionnaires have been published, the heterogeneity in their use makes it difficult to compare across multiple studies and in clinical practice (Caljouw et al., 2008; Sitzia, 1999; González et al., 2005; Chanthong et al., 2009). Thus, there is currently no “gold standard” to measure patient satisfaction. That being said, since CMS as well as commercial carriers formulate hospital reimbursement decisions based on the results of CAHPS surveys, special attention should be paid to these surveys although they may not be well validated scientifically.

### Patient satisfaction and clinical outcomes

The relationship of patient satisfaction and clinical outcomes is frequently debated due to the difficulty in conducting randomized controlled trials to test this association. Specifically, because it is not ethically possible to prospectively assign patients to a “satisfied” and a “dissatisfied” group, all studies on this topic are cross-sectional or observational in nature and therefore reflect association rather than causation. The most significant factor, in our opinion, is the cross-sectional nature of the studies within this space.

In general, higher patient satisfaction has been associated with positive clinical outcomes (Doyle et al., 2013; Larson et al., 1996). In a recent extensive review article on patient experience and clinical safety and effectiveness, Doyle and colleagues showed that of the 55 cited peer review studies, 77% showed a positive association (high patient satisfaction was related to clinical safety and effectiveness), 22% showed no association and < 1% showed a negative association (Doyle et al., 2013). Patients who were more satisfied with their medical care showed greater adherence to treatment plans (Bartlett et al., 1984; Haskard Zolnierek & Dimatteo, 2009), fewer hospital readmissions (Boulding et al., 2011), and a greater intention to keep follow-up appointments (Freed et al., 1998). And patients treated at hospitals with higher patient satisfaction scores experienced lower rates of post-operative mortality, death after any complication, and minor complications (Sacks et al., 2015).

Moreover, some studies have shown no association between patient experience and clinical outcomes (Fisher et al., 2003; Sequist et al., 2008; Werner & Bradlow, 2006), whereas others have reported poorer patient outcomes with higher experience scores (Fenton et al., 2012). For example, Sequist and colleagues showed no significant associations between patient experience and clinical outcomes in a study of 373 practice sites and 119 primary care physicians (Sequist et al., 2008). Fenton and colleagues linked higher patient satisfaction scores to higher mortality rates even after adjusting for factors such as chronic disease, socio-demographics, and availability to care (Fenton et al., 2012). Furthermore, physicians’ attempts to satisfy patients can lead to negative clinical outcomes. For instance, to minimize post-operative pain, pain relief may be achieved by overprescribing opioids. These well-intended actions contribute to opioid addiction, death from opioid overdose, and ultimately our current opioid epidemic (Bernard et al., 2018).

The various relationships between patient satisfaction and clinical outcomes could be explained by the fact that patient experience and clinical outcomes are complex concepts that include multiple sub-domains, and a wide range of factors contribute to its assessment as well as study design and analysis.

### Patient satisfaction and operational cost outcomes

The financial impact of patients’ satisfaction scores is an outcome that remains controversial although, as we indicated in the introduction, high patient satisfaction scores have direct and indirect financial benefits for hospitals. In a 2013 systematic review of 61 studies, 34% of articles reported a positive association (higher quality of care associated with a higher cost of care), 30% reported negative associations, and 36% reported no association (Hussey et al., 2013).

Under the CMS’s VBP program, hospitals are offered monetary incentives for higher HCAHPS scores. Tsai and colleagues found that patients who had surgery at hospitals rated higher in satisfaction (as measured by HCAHPS) cost less for Medicare than low-quality institutions (Tsai et al., 2016). Specifically, Medicare spent $2698 less on the average risk- and hospital-adjusted payment at 30 days for major surgeries such as coronary artery bypass grafting (CABG), pulmonary lobectomy, endovascular repair of abdominal aortic aneurysm, colectomy, or hip replacement. The most significant area in cost-reduction was on post-acute care and lower readmissions rates (Tsai & Orav, 2015).

Higher patient satisfaction has also been associated with reduced health care utilization by less frequent visits to the emergency room (Fenton et al., 2012). Indirectly, satisfied patients are more likely to refer family and friends to a hospital in which they have had a positive experience (Lee, 2008), producing more revenue. Conversely, some researchers have argued that there may be no or even negative effects of patient satisfaction scores on health care expenditures. For example, Fenton and colleagues reported that higher patient satisfaction was associated with higher inpatient utilization, greater total health care expenditures, and higher expenditures on prescription drugs (Fenton et al., 2012). The association of satisfied patient with the greater use of inpatient services and higher health care expenditures could be explained by increased likelihood that satisfied patients are more likely to seek health care. Overall, whether patient satisfaction scores contribute to health care expenditures is still up for debate.

## Discussion

### Can we predict patient satisfaction hospital scores?

Although we can debate on whether or not patient satisfaction impacts clinical or hospital costs, it is clear that CMS will penalize hospitals based on the VBP program if their patient satisfaction scores are low. As a result, it is critical to identify the determinants of patient satisfaction and direct various interventions based on these predictors. In examining the literature, one can see that identifying determinants of patient satisfaction scores is highly complex partially because of the paucity of clinical outcome data that are connected directly to each provider (Fig. 1).

### Modifiable factors: clinician communication skills

In the absence of such granular clinical data, patients tend to use proxies such as their providers’ communication skills when rating the quality of their medical care (Table 1). Specifically, patients place a high value on communication with healthcare providers within the perioperative settings (Hepner et al., 2004). For surgical patients, greater levels of detailed communication contribute to better patient satisfaction scores (Kahn et al., 2015). Patients highly value being treated with respect and knowing that providers are listening to what they have to say (Kahn et al., 2015). Satisfaction is also affected by the attention of staff to patient complaints (Gebremedhn & Lemma, 2017), prior explanation of diagnostic tests and procedures (Mira et al., 2009), and a surgeon’s effective communication pre-operatively and on the day of surgery (Tevis et al., 2015). The kindness and regard of caregivers in making patients feel safe improves patients’ perception of their surgical experiences (Capuzzo et al., 2007). Patients were dissatisfied with provider communication when they did not feel involved in the decision-making process and had poor continuity of care by the anesthesiologist (Heidegger et al., 2002).

**Table 1:** Studies findings on factors influencing patient satisfaction: clinician communication skills

Author (year)	Number of participants	Study design	Questionnaire	Main factors influencing patient satisfaction
Hepner et al. (Hepner et al., 2004) (2004)	857	Cross-sectional	Hospital-created for pre-operative clinic	Communication and information provided during pre-operative visit
Kahn et al. (Kahn et al., 2015) (2015)	182	Cross-sectional	HCAHPS	Respect from doctors, doctors listening, nurses’ listening, doctors’ explanations, and attempts to control pain
Gebremedhn et al. (Gebremedhn & Lemma, 2017) (2014)	269	Cross-sectional	Questions on various perioperative experiences	Patient admission status, information about the disease and operation, and operation theater staff attention to the patients’ complaints
Mira et al. (Mira et al., 2009) (2009)	23,438	Cross-sectional	SERVQUAL and additional questions on surgical experience	Information at admission, knowing what type of professional one was dealing with at any given time, informed consent, information about home care after discharge
Schmocker et al. (Tevis et al., 2015) (2015)	456	Cross-sectional	S-CAHPS	Surgeon’s preoperative communication and attentiveness on the day of operation
Capuzzo et al. (Capuzzo et al., 2005) (2005)	219	Cross-sectional	23-item instrument on patient satisfaction	Kindness and regard of caregivers, feeling safe, and information given by anesthetist
Heidegger et al. (Heidegger et al., 2002) (2002)	2348	Cross-sectional	Psychometric measure on anesthesia care	Information, involvement in decision-making and continuity of personal care by the anesthetist

*HCAHPS* Hospital Consumer Assessment of Healthcare Providers and Systems, *S-CAHPS* Surgical Consumer Assessment of Healthcare Providers and Systems

Good physician communication has been linked to many positive clinical outcomes such as greater treatment adherence (Bartlett et al., 1984; Haskard Zolnierek & Dimatteo, 2009), improved health outcomes (Kelley et al., 2014; Stewart, 1995), and decreased risk of malpractice allegations (Hickson et al., 2002). Unfortunately, physicians may have a different perception of the care they provide when compared to the patient’s experience (Olson & Windish, 2010). Some reports have indicated that many physicians are rated low in their patient communication skills (Marvel et al., 1999; McBride et al., 1994). With the introduction of HCAHPS, improvements have been made in the provider and patient communication (Boissy et al., 2016); however, continued improvements in this area would be beneficial due to the significant impacts on patient experiences.

### Information provision to patients

A major determinant of patient satisfaction across various inpatient and outpatient surgical specialties is information provided to patients (Table 2). There is a clear desire of information from patients (Caljouw et al., 2008; Mira et al., 2009; Lemos et al., 2009; Hawkins et al., 2012; Leinonen et al., 2001). Specific time points during the surgical experience in which information is provided are important and include the pre-operative visit, informed consent, surgical procedure episode, discharge, and postoperative care (Gebremedhn & Lemma, 2017; Mira et al., 2009; Fung & Cohen, 2001; Oswald et al., 2018). Patient satisfaction was higher when written information was supplemented with verbal information during the pre-operative visit (Johnson et al., 1999). In a cross-sectional study of 170 patients, more detailed information before surgery was found to increase patient anxiety (Forsberg et al., 2015). However, in randomized controlled trials of children undergoing surgery, more detailed pre-operative information was not found to increase child or parent anxiety (Kain et al., 1997; Inglis & Farnill, 1993). Patients dissatisfied with their surgical experience generally wanted more personalized information about the surgery, perioperative period, and home care instructions that is ideally provided in a format most appropriate to the patient educational level (Leinonen et al., 2001; Oswald et al., 2018; Forsberg et al., 2015; Otte, 1996).

**Table 2:** Studies findings on factors influencing patient satisfaction: information provision to patients

Author (year)	Number of participants	Study design	Questionnaire	Main factors influencing patient satisfaction
Lemos et al. (Lemos et al., 2009) (2009)	251	Cross-sectional	Questions on logistics, and those relating to surgery	At discharge: postoperative pain control, waiting time for surgery, patient changing room conditions 30 days post-surgery: clinical outcome, information given, and postoperative pain control
Mira et al. (Mira et al., 2009) (2009)	7899 inpatients, 15,539 outpatients	Cross-sectional	SERVQUAL and additional questions on surgical experience	Inpatient: information at admission, knowing what type of professional one was dealing with, and informed consent Outpatient: informed consent, information about home care after discharge
Caljouw et al. (Caljouw et al., 2008) (2008)	307	Cross-sectional	Leiden Perioperative care Patient Satisfaction questionnaire	How patients were approached and the amount of information they received
Leinonen et al. (Leinonen et al., 2001) (2001)	874	Cross-sectional	Modified Good Nursing Care Scale	Amount of information received and encouragement to ask more questions about unclear matters
Fung et al. (Fung & Cohen, 2001) (2001)	30	Cross-sectional	Questions pre-operative, intra-operative, pre-discharge and post-discharge outpatient anesthesia care	Information received and communication with the physician and staff members
Oswald et al. (Oswald et al., 2018) (2018)	292	Cross-sectional	EORTC-Info-25	Amount of information received
Johnson et al. (Johnson et al., 1999) (1999)	1445	Cross-sectional	Measures of relative satisfaction with various core aspects of care	Printed discharge information received
Forsberg et al. (Forsberg et al., 2015) (2015)	170	Cross-sectional	Patient’s Perspective questionnaire (QPP)	Personalized information about the surgery and perioperative period
Otte (Otte, 1996) (1996)	8	Qualitative	Semi-structured interviews on outpatient surgical experience	Amount of information received

*EORTC-QLQ* European Organization for Research and Treatment of Cancer Quality of Life Questionnaire

### Patient expectations and activation

Patient fulfillment of expectations is a predictor of satisfaction scores (Table 3) (Bjertnaes et al., 2012; Bleich et al., 2009). Consider a scenario in which two patients with the same diagnosis receive the same standard of care treatment, however one patient was anticipating an alternative treatment. Satisfaction scores of their health care encounters may differ. Additionally, patient activation can influence the patient experience. In this context, activation is described as being engaged and actively participating in one’s care. It involves a patient’s knowledge, skills, and willingness to manage their own health and care (Hibbard et al., 2004). An engaged patient is more likely to be more satisfied with the health care system as they may be more likely to ask questions to clarify their concerns or have a clear understanding of the reasons for their care. Greater activation can even improve health care outcomes (Hibbard & Greene, 2013).

**Table 3:** Studies findings on factors influencing patient satisfaction: patient activation and expectations

Author (year)	Number of participants	Study design	Questionnaire	Main factors influencing patient satisfaction
Bjertnaes et al. (Bjertnaes et al., 2012) (2012)	10,912	Cross-sectional	National patient-experience survey	Patient-reported experiences with nursing services, fulfillment of patient expectations, experiences with doctor services and perceived incorrect treatment
Bleich et al. (Bleich et al., 2009) (2009)	33,734	Cross-sectional	World Health Survey	Patient expectations, health status, type of care, and immunization coverage
Roseman et al. (Roseman et al., 2013) (2013)	–	Review	–	Patient activation and engagement in their care

### Patient’s reported symptoms

Post-operative events have been related to patients’ perioperative satisfaction. Specifically, greater satisfaction with outpatient surgery was reported when patients experienced less post-operative infection, inflammation, and pain (Gan et al., 2014; Lemos et al., 2009). In patients who underwent major orthopedic procedures, those who reported greater satisfaction experienced lower levels of pain and perceived that the physician and nurses showed concerned about their pain (Jamison et al., 1997). Patients who were less satisfied experienced persistent pain, nausea, vomiting, and other minor adverse outcomes post-operatively (Hickson et al., 2002; Bjertnaes et al., 2012; Bleich et al., 2009; Hibbard et al., 2004). Pain that was unexpected was associated with decreased satisfaction compared to expected pain (Bain et al., 1999). In addition, psychological distress such as anxiety during the perioperative period can influence post-operative pain and pain control (Perry et al., 1994; Vaughn et al., 2007). Clearly, post-operative symptoms are important to patients’ experiences and the management of emotional and physical symptoms is potential areas of improvement to increase patient perioperative satisfaction (Table 4).

**Table 4:** Studies findings on factors influencing patient satisfaction: patient’s reported symptoms

Author (year)	Number of participants	Study design	Questionnaire	Main factors influencing patient satisfaction
Lemos et al. (Lemos et al., 2009) (2009)	251	Cross-sectional	Questions on logistics, and those relating to surgery	Post-operative infection and/or inflammation, pain, and surgical outcome
Capuzzo et al. (Capuzzo et al., 2005) (2005)	219	Cross-sectional	23-item instrument on patient satisfaction	Pain at site of surgery, nausea, and vomiting
Jamison et al. (Jamison et al., 1997) (1997)	119	Cross-sectional	13-item measure on patient satisfaction	Post-operative pain, perception of physician and nurses concern about patient’s level of pain
Lehmann et al. (Lehmann et al., 2010) (2010)	12,276	Cross-sectional	Questions on perioperative minor adverse	Minor adverse events including nausea, vomiting, sore throat, hoarseness
Myles et al. (Myles et al., 2000) (2000)	10,811	Cross-sectional	Questions on postoperative outcomes	Postoperative pain, nausea and vomiting, and other complications
Bain et al. (Bain et al., 1999) (1999)	3408	Cross-sectional	Questions on information, outcomes, timing, and support services	Post-operative pain

### Physicians skills and behaviors

It is no doubt that physicians’ skills and behaviors contribute to patient satisfaction. Those which have been studied in the context of patient satisfaction include technical skills, pain management skills, and physicians’ respect to privacy.

In regards to technical skills, although most physicians tend to consider these skills to be a substantial contributor in patient satisfaction, it has been shown to not be a significant factor (Table 5) (Chung et al., 1999). This may be explained by the difficulties patients have in assessing this surgical skill, and the lack of public data to support this context. Furthermore, the management of postoperative pain can be assessed as a skill. Physicians who are more knowledgeable in pain management and who are better able to adequately control patients’ pain may have more satisfied patients (Meissner et al., 2015). Lastly, protecting patient privacy has been shown to be an important factor to patient satisfaction, particularly within the perioperative setting. When patient privacy and dignity are compromised, patients expressed feeling powerless, vulnerable, and anxiety (Rhodes et al., 2006). Educating and improving physician’s maintenance of patient modesty throughout the perioperative period can improve the patient experience. In total, there are multiple physician skills that if improved can enhance patient satisfaction.

**Table 5:** Studies findings on factors influencing patient satisfaction: skill of the physician

Author (year)	Number of participants	Study design	Questionnaire	Main factors influencing patient satisfaction
Chung et al. (Chung et al., 1999) (1999)	345	Cross-sectional	Visit Specific Patient Satisfaction Questionnaire (VSQ)	Not predicted by technical skills of physicians
Rhodes et al. (Rhodes et al., 2006) (2006)	–	Systemic Review	–	Maintaining patient privacy

### Operational function of the hospital

Important predictors of patient surgical experience include the organizational and structural components of the perioperative environment (Table 6), specifically, the nurse-to-patient ratio (Mazurenko et al., 2015), technical infrastructure (i.e., medical records system) (Mazurenko et al., 2015), and operations of admittance and discharge (Schoenfelder et al., 2010). In an outpatient plastic surgery clinic, patient satisfaction was significantly predicted by efficient clinic operations (e.g., scheduling of appointments and length of time to get an appointment) (Chung et al., 1999). Patients who experienced delays or had longer waiting times between admission, operation, and discharge were more likely to be dissatisfied with their surgical care (Bain et al., 1999; Fregene et al., 2017). Dissatisfied patients felt that there should have been more efficient scheduling and planning of their surgery (Otte, 1996). Patients who felt that their surgical unit was overcrowded were also less satisfied (Hart et al., 1996).

**Table 6:** Studies findings on factors influencing patient satisfaction: operational function of hospital

Author (year)	Number of participants	Study design	Questionnaire	Main factors influencing patient satisfaction
Bain et al. (Bain et al., 1999) (1999)	3408	Cross-sectional	Questions on information, outcome, and timing of events	Waiting times between admission, operation, discharge, and unexpected pain
Otte (Otte, 1996) (1996)	8	Qualitative	Semi-structured interviews	Efficiency of scheduling and planning of their surgery
Chung et al. (Chung et al., 1999) (1999)	345	Cross-sectional	Visit Specific Patient Satisfaction Questionnaire (VSQ)	Efficiency of clinic operation (e.g., scheduling of appointments and waiting time)
Mazurenko et al. (Mazurenko et al., 2015) (2015)	12	Qualitative	Interview on patient experience and satisfaction	Staffing (e.g., nurse to patient ratio), technical infrastructure (e.g., medical records system) and interdisciplinary relationships
Schoenfelder et al. (Schoenfelder et al., 2010) (2010)	2699	Cross-sectional	23 items on perceived care, demographics, and visit characteristics	Interpersonal manner of medical practitioners and nurses, organization of operations, admittance, and discharge, and perceived length of stay
Fregene et al. (Fregene et al., 2017) (2017)	n/a	Cross-sectional	Questions on overall satisfaction, fasting times and communication	Waiting times, communication and fasting
Hart et al. (Hart et al., 1996) (1996)	118	Cross-sectional	Questions on preoperative period, attitude of the personnel, and postoperative period	Overcrowded departments and lack of a sufficient number of registered nurses during night shifts

Patients also experience various stressors during hospital admissions that may affect their overall health care experience. A predictor of poor patient satisfaction is the development of the posthospital syndrome (PHS). PHS is described as a transient period of vulnerability after hospitalization where patients are at a higher risk for adverse events due to the experience of repetitive hospital-related stressors (Goldwater et al., 2018; Bueno et al., 2010; Drye et al., 2012). Common stressors include sleep disruptions from machine alarms or frequent health care provider examinations, painful stimuli from vital checks or procedures, and poor nutrition from withholdings of regular meals (Creditor, 1993). Indeed, a recent publication compared patient hospitalization to a painful stimulus animal model (Goldwater et al., 2018). As this animal model may closely resemble those experiences of hospitalized patients, reducing exposure to hospital stresses is warranted to enhance the patient experience.

### Interdisciplinary model of care

In a large study completed in 26 hospitals, one of the main predictors of patient satisfaction with their surgical experience was the perceived interpersonal manner of physicians and nurses (Table 7) (Schoenfelder et al., 2010). Many patients liked evidence of efficient communication between health care professionals, which they believe would prevent errors in their care (Lyndon et al., 2011). As an example, patients reported that they preferred instructions received from their hospitalist to align with that of their primary care physician (Mazurenko et al., 2015). A recently implemented interdisciplinary clinical model, the perioperative surgical home (PSH), was also described to enhance patient satisfaction (Kain et al., 2014). PSH is a patient-centered model that improves clinical pathways and reduces system-related variability in the surgical experience. Overall, improvements in the relationships between patient and provider as well as between hospital staff members can enhance patient experience and outcomes.

**Table 7:** Studies findings on factors influencing patient satisfaction: interdisciplinary model of care

Author (year)	Number of participants	Study design	Questionnaire	Main factors influencing patient satisfaction
Mazurenko et al. (Mazurenko et al., 2015) (2015)	15	Qualitative	Focused group interview	Interdisciplinary relationships, technical infrastructure, and staffing
Schoenfelder et al. (Schoenfelder et al., 2010) (2010)	2699	Cross-sectional	Questions perceived care, patient demographics, and visit characteristics	Interpersonal manner of medical practitioners and nurses, organization of operations, admittance, and discharge, and perceived length of stay

### Non-modifiable factors: demographic and health status predictors

The multifaceted nature of various non-modifiable factors associated with patient experience creates inherent challenges in measuring patient satisfaction scores (Table 8). It has been shown that older patients (variably defined as greater than 50, 65, or 70 years of age) are more likely to be satisfied with their surgical experience compared to younger patients (Caljouw et al., 2008; González et al., 2005; Mira et al., 2009; Capuzzo et al., 2007; Capuzzo et al., 2005; Hawkins et al., 2012; Danforth et al., 2014; Hall & Dornan, 1990; Teunkens et al., 2017; Maurice-Szamburski et al., 2013; Martin et al., 2011). This observation, however, is controversial and some studies have found no correlation with age and patient satisfaction in the surgical setting (Schoenfelder et al., 2010; Hamilton et al., 2013). Similarly, whereas some studies have found that men tend to be more satisfied with surgical care compared to women (Caljouw et al., 2008; Mira et al., 2009; Danforth et al., 2014; Teunkens et al., 2017; Maurice-Szamburski et al., 2013), other studies have found that variations in patient satisfaction with surgical services were not explained by gender (Mira et al., 2009; Schoenfelder et al., 2010; Hamilton et al., 2013). Mixed findings have also been demonstrated with regard to patients’ level of highest education. In a study regarding outpatient surgery, patients with a higher level of education are more likely to be satisfied (Teunkens et al., 2017). However, a mixed study with inpatient and outpatient surgery showed that in both populations, patients with a lower level of education were more satisfied (Mira et al., 2009). With regards to occupation, patients with paid employment were reported to be less satisfied with their perioperative experience compared to retired patients or those with household duties (Caljouw et al., 2008). Patient’s health status also has a wide range of effects on satisfaction in the surgical setting. One study reported that patients with a better health status had higher satisfaction scores (Capuzzo et al., 2007). However, other studies have shown that healthier patients were less satisfied with their surgical care and some have shown no correlation between the two variables (Lehmann et al., 2010; Danforth et al., 2014; Hamilton et al., 2013). Other non-modifiable factors reported in the literature include taking outpatient narcotics and admissions via the emergency department, both of which were related to lower satisfaction with inpatient surgical experience (Johnson et al., 1999; Danforth et al., 2014). Patients who traveled further (> 50 miles) for their surgery were more satisfied than patients who traveled less. Although we cannot alter non-modifiable factors like patient demographics, institutions need to practice caution when evaluating scores between populations that differ significantly with regard to such characteristics.

**Table 8:** Studies findings on factors influencing patient satisfaction: non-modifiable factors

Author (year)	Number of participants	Study type	Main factors associated with higher satisfaction (unless stated otherwise)					
			Age	Gender	Education	Occupation	Health status	Other
Hawkins et al. (Hawkins et al., 2012) (2012)	–	Systematic review	Older (age not specified)					
Capuzzo et al. (Capuzzo et al., 2005) (2005)	219	Cross-sectional	Older (> 55 years old)					
Auquier et al. (Auquier et al., 2005) (2005)	874	Cross-sectional	Older (> 65 years old)					
Martin et al. (Martin et al., 2017) (2017)	18,373	Retrospective	Older (age not specified)					
Maurice et al. (Maurice-Szamburski et al., 2013) (2013)	390	Cross-sectional	Older (> 55 years old)	Males				
Teunkens et al. (Teunkens et al., 2017) (2017)	5424	Cross-sectional	Older (age not specified)	Males	Higher education			
Mira et al. (Mira et al., 2009) (2009)	23,438	Cross-sectional	Inpatient: older (age not specified), Outpatient: younger (age not specified)	Inpatient: males, Outpatient: no difference	Lower education			
Caljouw et al. (Caljouw et al., 2008) (2008)	307	Cross-sectional	Older (> 50 years old)	Males		Household duties or retired (compared to employed)		
Capuzzo et al. (Capuzzo et al., 2007) (2007)	1290	Cross-sectional	Older (> 70 years old)				Higher health status	
Lehmann et al. (Lehmann et al., 2010) (2010)	12,276	Cross-sectional					Lower health status	
Danforth et al. (Danforth et al., 2014) (2014)	1340	Retrospective	Older (age not specified)	Males			Lower health status	Taking outpatient narcotics and ED admissions were less satisfied
Schoenfelder et al. (Schoenfelder et al., 2010) (2010)	2699	Cross-sectional	Not explained by age	Not explained by gender				
Hamilton et al. (Hamilton et al., 2013) (2013)	4709	Cross-sectional	Not explained by age	Not explained by gender			Not explained by health status	
Johnson et al. (Johnson et al., 1999) (1999)	1445	Cross-sectional						ED admissions were less satisfied
Abtahi et al. (Abtahi et al., 2014) (2015)	12,777	Retrospective	Older (age not specified)					Traveled a greater distance (> 50 miles)

### Interventions to improve patient satisfaction

#### Clinician communication skills

Various hospital intervention programs have been implemented to improve patients’ perioperative experiences. One training program taught anesthesiologists how to establish a welcoming atmosphere, elicit the patient’s concerns about anesthesia and surgery, demonstrate empathy verbally and non-verbally, involve the patient in decision-making, and conclude the visit by reassuring the patient of ongoing care. This communication skill training increased patient satisfaction and decreased patient anxiety with surgery (Harms et al., 2004). Many studies conducted in non-surgical settings have confirmed the relationship that improving physician communication and delivery of information improves patients’ experiences (Boissy et al., 2016; Bredart et al., 2005; Levinson & Lesser, 2010).

#### Information provision to patients

Provision of valuable and appropriately tailored preoperative information can facilitate patients’ active involvement in their care and can contribute to an increase in satisfaction. Various modalities of information provision to patients during the pre-operative visit have been attempted. Along with standard verbal information, the implementation of additional written information in the form of pamphlets has been shown to improve satisfaction (Angioli et al., 2014; Straessle, 2011). And the use of an informational website or a supplemental video and written brochure improve satisfaction scores (Snyder-Ramos et al., 2005; Hering et al., 2005). One intervention showed that providing detailed drug information leaflets for anesthetic drugs was not thought necessary by many patients but did not increase pre-operative state anxiety (Lee & Gin, 2005). Thus, providing surgical patients with more information than necessary through the use of multiple modalities is effective in improving satisfaction with their surgical experience and does not result in negative consequences.

#### Operational function of the hospital

In regard to hospital functioning, one intervention program staggered patient arrival times, had the first surgical patient of the day arrive earlier, assigned a single point of contact for patients, and informed patients of the possibility of a delay on the day of the surgery during pre-operative visits. These changes increased the percentage of satisfied patients and also led to shorter waiting times, better dissemination of information, and fewer patients reporting hunger or thirst (Fregene et al., 2017). Organizations have also provided more training for their staff members based on patient satisfaction results and trained nurse practitioners to complete all initial assessments, eliminating multiple providers and repeated medical questioning. These improvements enhanced patient satisfaction in domains of pre-operative experience, courtesy and efficiency of the clinic staff, and waiting time (Harnett et al., 2010).

#### Optimizing patient recovery

The Enhanced Recovery After Surgery (ERAS) protocol and the Perioperative Surgical Home (PSH) are innovative perioperative management strategies used to reduce the length of hospital stay, costs, and complications **(Kain et al.,** 2014**;** **Melnyk et al.,** 2011**)**. ERAS includes preoperative counseling, optimization of nutrition, scheduled analgesic regimens, and early mobilization. A systematic review completed in 2010 found that patients managed with ERAS reported reduced pain after surgery. However, it also found no difference in patient satisfaction after ERAS compared to the conventional recovery protocol (Khan et al., 2010). In another study, patient satisfaction as measured by Press Ganey was reported to improve significantly in patients following ERAS after colorectal surgery (Thiele et al., 2015).

The patient-centered approach of the PHS model aims to coordinate perioperative care within a multidisciplinary team (Kain et al., 2014). This model incorporates certain components of ERAS, but it is a broader concept. Studies have shown that patients who were treated with the PSH pathway had shorter lengths of hospital stays and lower unplanned hospital admissions compared with the standard pathway (Qiu et al., 2017). Although the result data is still emerging, ERAS and PSH are examples of effective interventions that can improve patient satisfaction in specific populations.

## Conclusion

We identified a number of variables that if modified can improve patient satisfaction scores and as such clinical outcomes and financial reimbursement of the hospital. These variables include clinician communication skills, information provision to patients, physician skills and behaviors, and a multidisciplinary patient care approach. Some non-modifiable predictors such as age and baseline health status were shown to impact patient satisfaction scores. Although these patient variables cannot be modified, it is important to understand the measured outcomes of a specific institution within this context. That is, organizations ideally should be compared to “like-organizations,” but unfortunately, this is not a common practice. Lastly, we have identified interventions that have been successful in improving patient satisfaction scores. Considering the multidimensional aspects of patient satisfaction, a team-focused approach should be implemented when attempting interventions to improve satisfaction. We suggest that readers engage in a discussion with patient experience leaders in their own organizations and understand how they can enhance patient satisfaction scores while modifying practices and behaviors that are in their own control.

## Data Availability

All data generated or analyzed during this study are included in this published article.
